# Downregulation of the evolutionary capacitor Hsp90 is mediated by social cues

**DOI:** 10.1098/rspb.2015.2041

**Published:** 2015-11-22

**Authors:** Robert Peuß, Hendrik Eggert, Sophie A. O. Armitage, Joachim Kurtz

**Affiliations:** Institute for Evolution and Biodiversity, University of Münster, Hüfferstrasse 1, Münster 48149, Germany

**Keywords:** Hsp90, *Tribolium castaneum*, canalization, immune defence, stress, heat shock proteins

## Abstract

The relationship between robustness and evolvability is a long-standing question in evolution. Heat shock protein 90 (HSP90), a molecular chaperone, has been identified as a potential capacitor for evolution, since it allows for the accumulation and release of cryptic genetic variation, and also for the regulation of novel genetic variation through transposon activity. However, to date, it is unknown whether Hsp90 expression is regulated upon demand (i.e. when the release of cryptic genetic variation is most needed). Here, we show that Hsp90 has reduced transcription under conditions where the mobilization of genetic variation could be advantageous. We designed a situation that indicates a stressful environment but avoids the direct effects of stress, by placing untreated (focal) red flour beetles, *Tribolium castaneum*, into groups together with wounded conspecifics, and found a consistent reduction in expression of two Hsp90 genes (*Hsp83* and *Hsp90*) in focal beetles. We moreover observed a social transfer of immunity in this non-eusocial insect: there was increased activity of the phenoloxidase enzyme and downregulation of the immune regulator, *imd*. Our study poses the exciting question of whether evolvability might be regulated through the use of information derived from the social environment.

## 1. Introduction

Darwinian selection acts on phenotypes, but adaptive evolution results from changes in the genotypic composition of a population [1,2]. However, phenotypes often develop in a remarkably robust manner, even when facing environmental or genetic perturbations, a phenomenon known as canalization [3]. A central question in evolutionary biology is therefore what the links are between phenotype and genotype, and whether these links are constant or open to modifications [4–7]. Heat shock proteins (HSPs) are important mediators between the genotype and the phenotype; they are essential molecular chaperones, controlling the folding and degradation of proteins [8–10]. During periods of stress, competition for HSPs by damaged proteins reduces their availability. Genetic diversity in the population that codes for differences in protein sequences, which may normally be masked by HSPs (i.e. cryptic genetic variation), may then be translated into phenotypic differences upon which selection could act [11,12]. Via this buffering hypothesis, HSP90, which mainly interacts with signal transducers and developmental regulators [13], can be envisaged as an evolutionary capacitor, allowing genetic variation to be hidden from, or visible to, selection [13,14]. It is noteworthy that the cryptic genetic variation contains not just random variants; they could be, or have been, successful under certain ecological conditions. So whether the release of cryptic variation in a stressful environment is negative or positive is also dependent on the environment. A second hypothesis for how HSP90 could affect evolvability is through the suppression of transposons: under HSP90 depletion novel genetic diversity might be produced by the mutagenic effect of transposons [15]. Both the buffering and transposon suppression hypotheses place HSPs as key regulators of robustness and evolvability. However, there are differences in these two hypotheses: while the integration of transposons is a random mutagenic effect, the release of cryptic genetic variation has the potential to generate phenotypes that might already have proved successful under certain environments.

Wild *Drosophila melanogaster* populations show natural variation in the amino acid sequence and expression of the Hsp90 gene *Hsp83* [16,17], which suggests that HSP90 function itself might be subject to evolutionary adaptation [18,19]. Moreover, Hsp90 expression is upregulated when animals are directly subjected to ecologically relevant stressors [14], a situation that is in itself predicted to lead to a reduction of available HSP90 protein, due to increased competition among its binding partners for this chaperone (capacitor hypothesis; for review see [20]). It is difficult to disentangle this hypothesized consequence of reduced HSP90 availability (and thus the release of cryptic genetic variation) from the direct stressor effects, which would be necessary to support this important aspect of the capacitor hypothesis [13]. To our knowledge, it is unknown whether Hsp90 expression could also be regulated by ecologically relevant cues acting as an anticipated environmental stress, but independently of a direct stressor effect. Why might this be important? In a risky environment, a reduction of Hsp90 expression may be beneficial by mobilizing genetic variability, translating into more variable offspring whereby some might achieve higher fitness under adverse conditions, affording the parents a bet-hedging strategy.

Here, we tested whether Hsp90 expression can be regulated by environmental cues by performing two experiments in the red flour beetle, *Tribolium castaneum*. The first was designed to test whether cohabitation for 6, 12 or 18 h of focal unmanipulated beetles with wounded beetles resulted in regulation of *Hsp83*. We predicted a downregulation of *Hsp83* so as to mobilize genetic variability. We also measured the expression of well-described stress genes (*Hsp68*, *CytP450*) to monitor the stress level of the focal beetles. *Hsp68* is a gene of the Hsp70 gene family and is normally involved in the acute stress response (e.g. wounding and heat stress [21]). The CytP450 gene family has many functions, one of which is related to the stress response in *T. castaneum* [21]. We further hypothesized that wounding of conspecifics might lead to social transfer of immunity (i.e. enhanced immunity of group members), as has previously been shown in terms of enhanced protection against parasites in eusocial insects [22–24], but has not, to our knowledge, been found in relation to only wounding. Therefore, we investigated immune gene expression (*Att2*, *Col1*, *Thau*, *Imd* [21]), and additionally an individual trait and a social immune trait, haemolymph phenoloxidase (PO) and quinones secreted into the environment, respectively. In the first experiment, we used mixed sex cohabitation groups. Therefore, to ensure that the results we observed in the first experiment were not explained by responses to mating, in the second experiment we used a similar experimental set-up focusing on one cohabitation duration (18 h), and tested both mated and virgin beetles for stress and immune gene expression.

## 2. Material and methods

### (a) The model system and production of experimental individuals

The red flour beetle, *T. castaneum*, is a well-established model system to investigate behaviour, genetics, ecology and immunology [25]. Here, we used the Cro1 strain, which was collected in Croatia in 2010 [26] and allowed to adapt to laboratory conditions for at least 20 generations prior to the start of our experiments. Stock animals were maintained as non-overlapping generations in organic white flour type 550 supplemented with 5% yeast at 30°C and 70% humidity in a 12 L : 12 D cycle. Experimental individuals were kept under similar environmental conditions, except where stated otherwise. Experimental individuals were produced by allowing approximately 500 two-week-old adults to lay eggs in flour with 5% yeast for 24 h. The eggs were removed from the flour by sieving with a mesh size of 280 µm and maintained in a plastic box in 100 g of flour with 5% yeast for 10 days. After this time, the larvae were removed from the flour by sieving and individualized in 96-well plates, each containing 0.08 g flour with 5% yeast. The plates were sealed with clear tape, through which air holes were punctured for each well. Individualization was not done at an earlier stage because the eggs and early instar larvae are difficult to manipulate without damaging them.

### (b) Nomenclature of HSP90 genes

For abbreviations of the HSP90 genes and proteins, we used the following typographic rules: HSP90 refers to the protein in vertebrates and invertebrates, Hsp90 refers to the gene family of HSP90, and *Hsp83* refers to a specific gene of the Hsp90 gene family.

### (c) Experiment 1: Hsp90 gene expression and immunity in the presence of wounded conspecifics

#### (i) Experimental design

One week after adult eclosion, we randomly assigned beetles to one of four treatment groups. Each group consisted of 24 focal beetles and 24 non-focal marked beetles in 4.8 g of flour per glass jar (diameter 85 mm), which corresponds to a density of one beetle per 2.68 mm^2^. The non-focal beetles were marked on the pronotum with a small dot of white enamel paint. The four treatments were as follows, with the order of focal beetle/non-focal marked beetle (the abbreviation for the treatment group name is given in parentheses): (i) naive/naive (N^N^) was used as our control group; (ii) naive/wounded (N^W^); (iii) wounded/naive (W^N^); (iv) wounded/wounded (W^W^) (for experimental set-up, see electronic supplementary material, figure S1). Directly before cohabitation, beetles were wounded by pricking with a sterile dissection needle (diameter 10 µm) between the pronotum and occiput as described in [27]. Each treatment group was replicated four times for three different cohabitation periods: 6, 12 and 18 h (see electronic supplementary material, figure S1). It is known that immune and stress gene modulation occurs quickly, and therefore we looked at the acute response phase, as also examined by Behrens *et al.* [28] and Altincicek *et al.* [21]. To test whether the environment affected HSPs, stress and immunity, we quantified the gene expression of a set of relevant genes. Furthermore, we examined changes in the immune system by measuring the activity of the enzyme PO. We also measured the levels of an external immune defence: hydroquinone and benzoquinone levels in the flour. *Tribolium castaneum* exhibits intraspecific cannibalism [29]; however for this and the following experiment, all animals that were used in the cohabitation assay survived the cohabitation without any cannibalism.

#### (ii) Expression of immunity and stress-related genes

To examine whether there were gene expression changes in immunity upon our cohabitation assay, we chose to test the expression of *Imd*, which plays a signal transduction role in the immune deficiency (*imd*) pathway in insects (for review, see [30]). We also tested expression of the two antimicrobial peptides (AMPs) *Attacin2* (*Att2*) and *Coleoptericin1* (*Col1*), as well as *Thaumatin1* (*Thau*). AMPs are small cationic peptides that insert into and disrupt microbial membranes, thereby killing and clearing pathogens [31]. After infection, they are synthesized de novo and then released into the insect haemolymph by haemocytes and to a greater extent the fat body [31,32]. They have previously been found to be upregulated in response to bacteria as well as wounding in *T. castaneum* [21,28]. The antifungal protein *Thaumatin1* is also strongly upregulated upon infection or sepsis [18]. To test whether there were gene expression changes in stress-related genes, we examined *cytochrome P450* (*CytP450*), *Hsp90* (experiment 2 only), *Hsp83* and *Hsp68* [21,33–35]. HSPs are a well-known class of proteins involved in the stress response of insects, which act mostly as chaperones [9,10,13]. *Hsp68* and *CytP450* were also shown to be differently regulated upon different stressors (e.g. heat or wounding) in *T. castaneum* [21]. For each time point and experiment, 10 randomly chosen focal beetles of each group and replicate were pooled into a 1.5 ml microcentrifuge tube and frozen in liquid nitrogen. For total RNA extraction, frozen beetles were homogenized over liquid nitrogen with a sterile pestle, then 500 µl of Trizol (Ambion RNA) were added to each sample. The samples were further lysed by incubation at room temperature for 10 min with vortexing every 2 min. After centrifugation (18 000*g* at 4°C, 5 min), the supernatant was transferred to a new tube, and 100 µl chloroform was added and incubated at room temperature for an additional 15 min. The samples were centrifuged for 15 min at 11 500*g* and 4°C, and the upper aqueous phase was transferred to a new tube. For purification of the total RNA from the aqueous phase, we used the SV Total RNA Isolation System (Promega) according to the manufacturers' protocol, which included a DNase digestion step. After purification, the RNA concentration was measured using a NanoPhotometer Pearl (Implen, Germany). Next, 100 ng purified total RNA from each sample was used in a reverse transcription reaction using the SuperScript III (Invitrogen by Life Technologies GmbH) with random hexamer primers according to the manufacturer's protocol. The resulting cDNA was used undiluted for RT-qPCR analyses using immune and stress gene-specific primers (see electronic supplementary material, table S1), which were designed so that when the gene contained more than one exon, the primer crossed an intron–exon boundary. The amplification efficiencies (*E*) of the primers were determined with five dilutions (undiluted, 1 : 10, 1 : 100, 1 : 1000, 1 : 10 000) of template cDNA, where *E* = 10^−1^/slope (see electronic supplementary material, table S1). RT-qPCR was performed in a 96-well plate format, with a total reaction volume of 15 µl in each well. From each cDNA sample, two technical replicate qPCR reactions were performed using the Kapa Sybr Fast qPCR Mastermix for LightCycler 480 (Peqlab Biotechnologie GmbH) according to the manufacturer's instructions. The reaction was run on a LightCycler 480 (Roche) using the following protocol: 95°C for 5 min, followed by 40 cycles of annealing and amplification at 60°C for 1 min and denaturation at 95°C for 15 s. As a final step, the products were heated up to 95°C with continuous fluorescence measurements to obtain the melting curves, and subsequently cooled to 40°C. The resulting *Cp* values were calculated with the LightCycler 480 software using the second derivative maximum method [36] and expression differences between groups were calculated according to Pfaffl [37]:
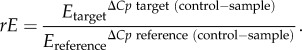


There relative fold expression differences (*rE*) between N^N^ (*control*) was tested in turn against each of the other three treatment groups (*sample*) (i.e. N^W^, W^N^ and W^W^). *E* indicates efficiency, *target* indicates the gene of interest (e.g. *Hsp83*) and *reference* indicates the geometric mean of two reference genes, *rp49* and *rpL13a* (see electronic supplementary material, table S1).

#### (iii) Individual immunity: phenoloxidase activity

PO activity is an important enzyme in insect immunity and is correlated with responses to infection, invasion and wounding [38]. This enzyme is synthesized in haemocytes as an inactive zymogen, pro-phenoloxidase (proPO), which can be activated by proteolytic cleavage [39]. We quantified the activity (*V*_max_) of PO to determine the immune system's activity. Therefore, five focal beetles from each jar of every treatment and replicate were randomly sampled after cohabitation for the respective time periods. The haemolymph was collected by puncturing the pleural membrane between the pronotum and occiput with a sterile hypodermic needle. The out-flowing droplet of haemolymph was collected in a sterile, pre-chilled glass capillary and transferred into 20 µl of pre-chilled Bis–Tris buffer [40]. To ensure that every sample contained a concentration of 0.05 µl haemolymph to 20 µl Bis–Tris buffer, we measured the volume of haemolymph extracted from each beetle (the volume was between approx. 0.05–0.1 µl per beetle) and subsequently adjusted the amount of Bis–Tris accordingly. Due to handling error, seven of the 240 samples were lost. To determine PO activity, 50 µl of distilled water and 50 µl Bis–Tris were added to a 96-well flat-bottomed plate with 20 µl of the haemolymph/Bis–Tris mixture. As a negative control, we added 20 µl Bis–Tris buffer without haemolymph. After adding 50 µl of l-Dopa (4 mg ml^−1^ l-Dopa dissolved in Bis–Tris) (Sigma-Aldrich), the absorbance was measured on a Tecan Infinite M200 plate reader at 490 nm and 37°C, with readings taken once every minute for 90 min. PO activity was measured as the 15 min window in the enzyme reaction when the enzyme kinetics showed a linear change in absorbance (*V*_max_). PO activity thereafter was calculated as *V*_max_ (sample) − *V*_max_ (blank).

#### (iv) External immunity: hydroquinone and benzoquinone levels in the flour

A phenomenon shared by eusocial insects and the group-living flour beetles is the alteration of the environment by the secretion of defensive chemical compounds [41–43]. *Tribolium castaneum* as well as *Tribolium confusum* secrete quinones (benzoquinone and hydroquinone) into their surroundings to protect their environment against pathogens [42,43]. Quinones have broad antimicrobial functions and can be regarded as an external immune trait [44]. We quantified the hydroquinone and benzoquinone levels that the beetles had secreted into the flour at 6, 12 and 18 h after cohabitation. Once the beetles had been removed from the flour, it was stored at 7°C in the dark for four weeks [44]. Storing the used flour under these conditions modifies the quinones into benzo- and hydroquinones, which can then be measured using a UV spectrophotometer plate reader assay at the given light spectra [44]. From each jar, 0.2 g flour was transferred to a 1.5 ml microcentrifuge tube containing 600 µl acetonitrile and kept at 4°C for 24 h. The tubes were then centrifuged for 5 min at 4°C and 11 000*g*. We then transferred 120 µl of the supernatant from each sample to a 96-well quartz plate (Hellma, Müllheim, Germany) and the peak height of hydroquinone and benzoquinone was measured in a Tecan Infinite M200 plate reader at 246 nm and 274 nm, respectively. A sample of flour that had not been exposed to beetles was used as a negative control, therefore the absorbance value for both quinones was determined by subtracting the corresponding value for the negative control.

### (d) Experiment 2: the effect of cohabitation on Hsp90 gene expression in relation to sex and mating status

#### (i) Experimental design

Here, we used male and female adult beetles that were either virgin or allowed to mate before cohabitation. Therefore, when the individualized larvae (as described above) became pupae, we determined the sex of each and thereafter individualized them again in new 96-well plates. One week after adult eclosion, half of the beetles were randomly allocated to the virgin treatment and therefore remained in the 96-well plates. The other half of the beetles were allocated to the mating treatment group, whereby 24 male and 24 female randomly selected virgin beetles were placed together for 24 h in a jar filled with 4.8 g flour with 5% yeast (see electronic supplementary material, figure S2, for the experimental set-up). We produced 12 replicate jars in this manner. This treatment allowed the beetles to mate in a group setting, similarly to experiment 1. We did not observe individual matings, therefore this set-up does not guarantee that every beetle had mated. However, given that *T. castaneum* is highly polygamous, males can mate with up to seven virgin females in 15 min [45], males often mount immediately after making contact with a female [46], and that males and females were cohabited for 24 h, it is likely that all of the beetles had copulated during this period. After 24 h of being allowed to mate, the beetles from the 12 jars were pooled together, and males and females were separated from each other. We then produced eight single-sex treatment groups: four groups consisted of the beetles that had been allowed to mate (i.e. (i) mated female N^N^, (ii) mated female N^W^, (iii) mated male N^N^ and (iv) mated male N^W^) and four treatment groups consisted of virgins (i.e. (v) virgin female N^N^, (vi) virgin female N^W^, (vii) virgin male N^N^ and (viii) virgin male N^W^) (electronic supplementary material, figure S2). The marking and wounding of the beetles was done as described for experiment 1. Each single-sex group consisted of 10 focal beetles and 10 marked beetles in a small Petri dish (diameter 60 mm) with 2 g of flour and 5% yeast, and each group was replicated seven times. The density was similar to experiment 1, allowing the same interaction space (2.68 mm^2^). The cohabitation time was 18 h. For this experiment, we examined only gene expression, and it was performed as described for experiment 1.

### (e) Statistical analyses

The PO and quinone data were analysed using JMP v. 9.0.0 for Macintosh. For PO activity in experiment 1, we performed a mixed-effects model (REML), with the response variable as Box–Cox transformed PO activity (*n* = 233 animals), and the fixed factors as time (6, 12 or 18 h), focal animal treatment (naive or wounded) and non-focal animal treatment (naive or wounded), including all of the possible interaction terms. The jar in which the animals had been held for cohabitation was included as a random factor (*n* = 48 jars). The quinone data could not be transformed to a normal distribution and had unequal variances. We therefore performed non-parametric Kruskal–Wallis tests for each of the quinones, and tested time (6, 12 or 18 h) and treatment (treatment groups 1–4) as fixed factors in separate models for each quinone (*n* = 47; one N^N^ sample (6 h) did not contain measurable amounts of quinones). The response variables were the hydroquinone or the benzoquinone levels.

The expression levels of each gene was calculated with Relative Expression Software Tool (REST 2009 [47]), based on the primer efficiencies (see electronic supplementary material, table S1) and overall maximal/minimal *Cp* values providing the variance of each primer product across all samples and pairwise comparisons against the control treatment (N^N^). REST performs a pairwise fixed reallocation randomization test to examine whether there are significant differences between the two groups. We allowed 2000 random reallocations of the observed *Cp* values to the two groups being tested; REST notes the expression ratio change for each reallocation, and the proportion of these effects gives the *p*-value assuming a two-sided test [47]. In our figures, we present the mean and standard errors as calculated according to the REST software (i.e. the results of the 2000 random reallocations). To correct for multiple comparisons, we used the false discovery rate (Benjamini Hochberg correction [48]).

## 3. Results and discussion

### (a) Hsp90 downregulation in the presence of wounded conspecifics

**Figure 1.**
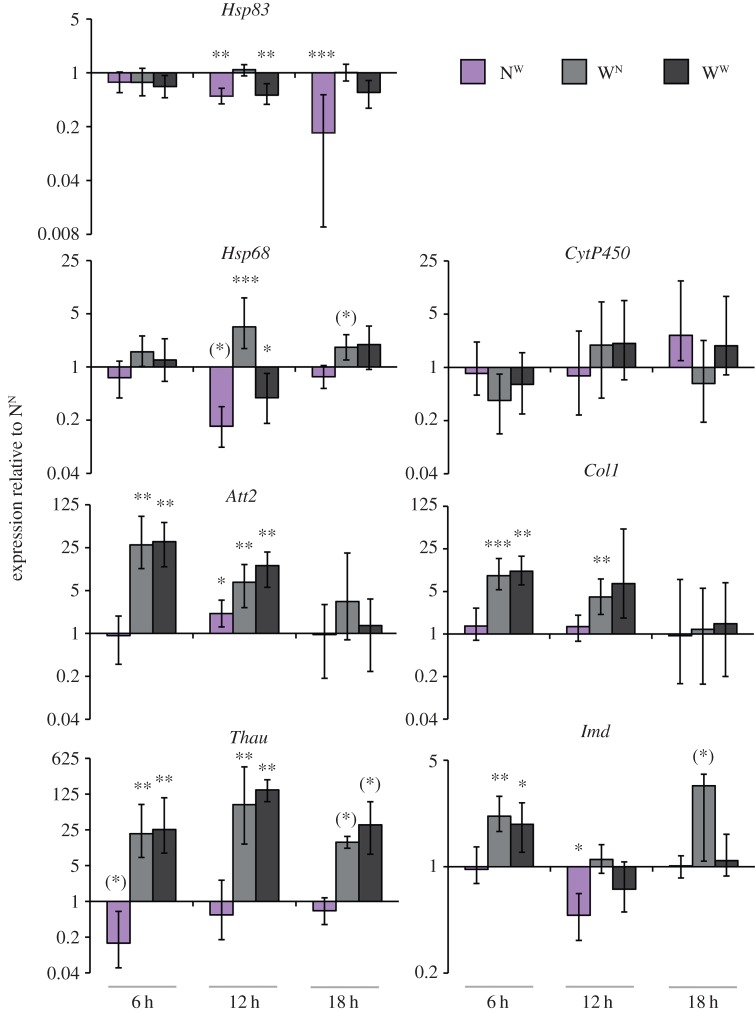
Gene expression of naive and wounded *T. castaneum* beetles after 6, 12 or 18 h of cohabitation with naive or wounded beetles. Naive focal beetles were kept together with wounded non-focal beetles (purple: N^W^), and wounded focal beetles were similarly kept with naive non-focal beetles (light grey: W^N^) or wounded non-focal beetles (dark grey: W^W^). We examined gene expression of HSPs, stress and immunity genes using RT-qPCR after 6, 12 and 18 h of cohabitation (for detailed experimental set-up, see electronic supplementary material, figure S1). Gene expression for each of the three treatment groups is given relative to the control N^N^ treatment group (i.e. naive focal beetles cohabited with naive non-focal beetles). Each bar is the mean of four biological replicates and each one contained total RNA of 10 focal animals. Means and error bars were calculated by REST 2009. Error bars indicate 1 s.e. Statistical significances are given after Benjamini Hochberg (FDR) corrections have been applied: *p ≤ 0.05, **p ≤ 0.01, ***p ≤ 0.001. Means that were significant before FDR are indicated with asterisks within parentheses.

We analysed the expression of the Hsp90 gene, *Hsp83*, in focal animals that were naive but had been cohabited with wounded conspecifics (N^W^; figure 1). After both 12 and 18 h of cohabitation, *Hsp83* expression was significantly decreased compared with the control group (figure 1), which were naive focal beetles that had been placed into groups with naive beetles (N^N^). As additional controls, we tested beetles that were wounded themselves and placed together with naive or wounded beetles (W^N^ and W^W^, respectively). *Hsp83* expression was also significantly decreased in the W^W^ group after 12 h of cohabitation, but not in the W^N^ group (figure 1), indicating that wounding as such did not result in any decrease of *Hsp83* expression (group W^N^). Wounding-associated direct stress might arguably rather shift the *Hsp83* expression level upwards, such that the overall result depends on the proportion of wounded beetles in the surrounding environment in combination with individual wounding.

Since we did not wound the focal beetles directly, we did not expect any direct physiological effect on stress genes. The expression of the stress-related gene *Cytochrome P450* (*CytP450*) was, as predicted, unchanged in focal N^W^ beetles (figure 1). By contrast, *Heat shock protein 68* (*Hsp68*), which is a gene of the stress-inducible HSP 70 group [21], showed a trend for downregulation at 12 h post-cohabitation in N^W^ as well as in W^W^ beetles, whereas W^N^ beetles showed *Hsp68* upregulation potentially as a direct result of wounding.

### (b) Social transfer of immunity

As predicted, the immune effector genes *Attacin 2*, *Coleoptericin 1* and *Thaumatin1* (*Thau*) were upregulated as a direct result of wounding (groups W^N^ and W^W^; figure 1). Consistent with social transfer of immunity, N^W^ showed a significant but weak upregulation of *Att2*. We moreover observed a weak downregulation of the key immune regulator *Immune deficiency* (*Imd*) at 12 h. It is noteworthy that *Imd* mutant *D. melanogaster* show higher resistance against UV stress based on suppression of apoptosis [49].

**Figure 2.**
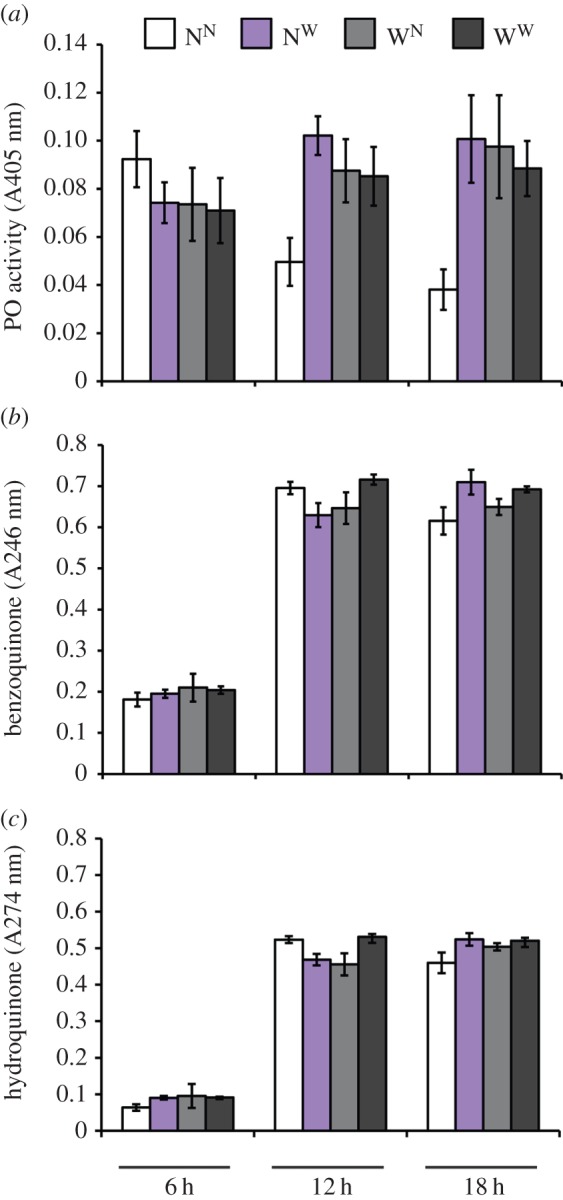
PO activity and secreted quinone levels of naive and wounded *T. castaneum* beetles after 6, 12 or 18 h of cohabitation with naive or wounded beetles. Naive focal beetles were allowed to cohabit for 6, 12 or 18 h with naive non-focal beetles (white: N^N^) or wounded non-focal beetles (purple: N^W^), and wounded focal beetles were similarly kept with naive non-focal beetles (light grey: W^N^) or wounded non-focal beetles (dark grey: W^W^). After this time, we measured internal personal immunity as (*a*) PO activity of haemolymph; each bar is the mean of all animals (*n* = 20) used (i.e. haemolymph from five beetles for each of the four biological replicates). We measured social immunity as the (*b*) benzoquinone and (*c*) hydroquinone levels in the flour that the beetles had been living in for the 6, 12 or 18 h cohabitation periods; in this case, each mean and standard error is calculated from four biological replicates (for detailed experimental set-up, see electronic supplementary material, figure S1). All error bars show 1 s.e.

Enzymatic activity of PO, a key component of insect immune defence [50] showed that there was a significant interaction between the focal and the cohabitant treatment (table 1 and figure 2*a*). Individual contrasts showed that as predicted, the focal animals that were wounded themselves (W^N^ and W^W^) had higher PO than the N^N^ group (N^N^ versus W^N^: *t* = −3.134, *p* = 0.0034; N^N^ versus W^W^: *t* = −2.953, *p* = 0.0055), but of particular interest was the fact that N^W^ beetles had increased PO compared with N^N^ (*t* = −4.654, *p* < 0.0001), illustrating that being kept with wounded conspecifics is sufficient for unwounded beetles to increase activity of an immune enzyme. Indeed, there was also an overall increase in PO when individuals were kept with wounded conspecifics (table 1). Taken together, the immune gene expression and PO data show a clear signal of social transfer of immunity to the non-wounded beetles. This effect is noteworthy, since it is, to our knowledge, the first demonstration of such an effect of social transfer of immunity (i.e. increased immunity in naive beetles after living with wounded conspecifics) in an insect that is not eusocial, but instead lives in large aggregations of related and unrelated animals.

**Table 1.** The effects of focal treatment (naive or wounded), cohabitant treatment (naive or wounded) and time of cohabitation (6, 12 or 18 h) on PO activity of focal beetles. *p-*values less than 0.05 are in italic type.

tested effect	d.f.	*F*-ratio	*p*-value
focal treatment	1,36.22	1.042	0.314
cohabitant treatment	1,36.22	9.917	*0.003*
time	2,36.22	0.120	0.896
focal treatment × cohabitant treatment	1,36.22	11.83	*0.002*
focal treatment × time	2,36.22	2.861	0.070
cohabitant treatment × time	2,36.22	1.555	0.225
focal treatment × cohabitant treatment × time	2,36.22	1.200	0.313

In addition to internal (personal) immunity [24], adult flour beetles release quinone secretions into the surrounding environment, thereby controlling environmental microbiota [44,51,52]. Since the molecular pathways of internal immunity (PO) and external immunity (quinones) interact with one another [44], we hypothesized that PO and quinones might be correlated. Moreover, *T. castaneum* could secrete quinones into the flour upon wounding and thereby transfer the information of the wounding. The levels of benzoquinone (figure 2*b*) and hydroquinone (figure 2*c*) in the flour in which the beetles had been cohabiting increased with time: a significant increase occurred between 6 and 12 h of cohabitation and no further increase occurred between 12 and 18 h (table 2). Unlike the internal immune traits, there was no effect of cohabitation treatment on the quinones (table 3). This suggests that information about wounding, and thereby the effects of the social environment on *Hsp83* expression and immunity, are probably not a consequence of quantitative changes in environmental quinone levels.

**Table 2.** Test for the main effect of time of cohabitation on benzoquinone and hydroquinone levels in the flour, as well as the individual comparisons between time points for each measured quinone. *p-*values less than 0.05 are in italic type.

	benzoquinone (A246 nm)				hydroquinone (A274 nm)			
tested effect	d.f.	*χ*^2^-value	*z*-value	*p-*value	d.f.	*χ*^2^-value	*z*-value	*p-*value
time (main effect)	2	30.05	** **	<*0.0001*	2	30.14		<*0.0001*
6 versus 12 h			4.724	<*0.0001*			4.724	<*0.0001*
6 versus 18 h			4.724	<*0.0001*			4.724	<*0.0001*
12 versus 18 h			0.283	0.7777			−0.528	0.598

**Table 3.** Test, within time of cohabitation, for the effect of combinations of individual and cohabitation treatments (i.e. whether there is a difference between N^N^, N^W^, W^N^ and W^W^) on quinone levels in the flour. The *p-*value less than 0.05 is in italic type; none of the groups were statistically significantly different from the control group (N^N^) in multiple comparisons.

	benzoquinone (A246 nm)			hydroquinone (A274 nm)		
tested effect	d.f.	*χ*^2^-value	*p-*value	d.f.	*χ*^2^-value	*p-*value
treatment (6 h)	3	6.040	0.110	3	1.454	0.693
treatment (12 h)	3	9.383	0.025*	3	5.757	0.124
treatment (18 h)	3	3.640	0.303	3	5.052	0.168

### (c) The downregulation of Hsp90 genes is independent of sex and mating status

**Figure 3.**
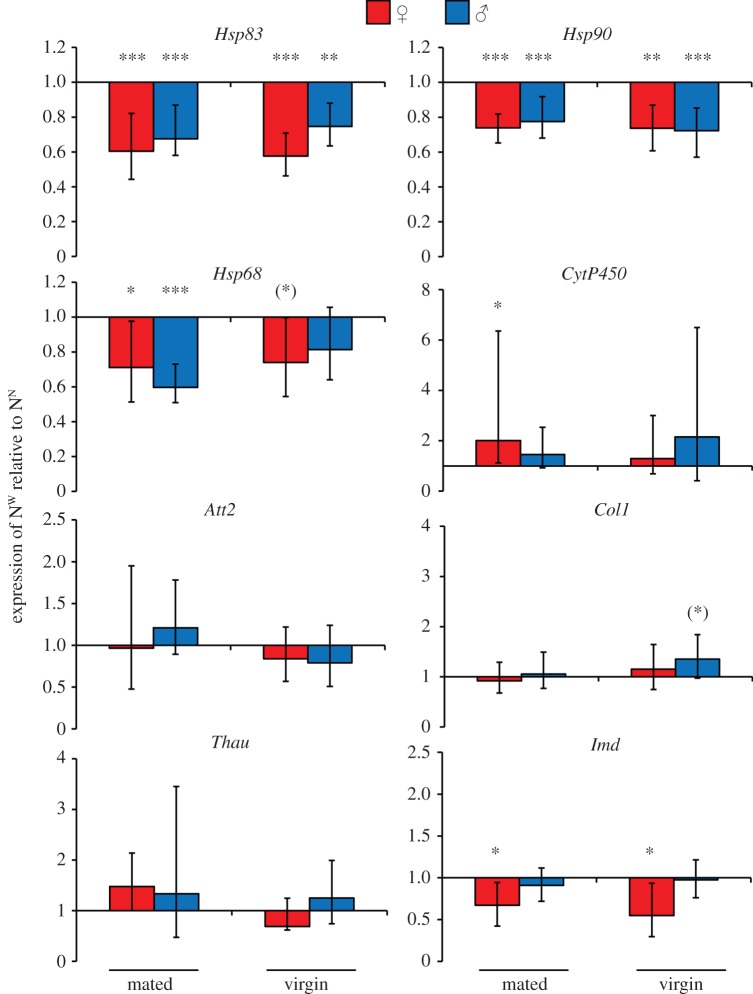
Gene expression of mated or virgin naive focal *T. castaneum* beetles that were cohabitated for 18 h with wounded beetles of the same sex and mating status. Prior to cohabitation, beetles were allowed to mate or kept individually. Beetles were then wounded or remained naive and placed into cohabitation groups. Eight cohabitation groups were formed. To produce the N^W^ groups, mated naive females or males were placed with mated wounded females or males, respectively (N^W^). Two similar groups were set up for virgin females and males. The four control groups were the same except the cohabited beetles were always naive (N^N^). Gene expression of the four N^W^ groups is shown relative to the corresponding four N^N^ control groups. Each bar is the mean of seven biological replicates, and for each sample total RNA of 10 animals was used (for detailed experimental set-up, see electronic supplementary material, figure S2). Means and error bars were calculated by REST 2009. Error bars indicate 1 s.e. Statistical significances after Benjamini Hochberg (FDR) corrections are indicated with: *p ≤ 0.05, **p ≤ 0.01, ***p ≤ 0.001. Means that were significant before FDR are indicated with asterisks within parentheses.

Up to this point, we used mixed-sex groups, therefore we reasoned that one explanation for the effects of cohabitation could have been that wounding influences mating behaviour, and it is this that affected *Hsp83* expression. Moreover, *Hsp83* expression might differ between males and females. To disentangle mating effects from cohabitation effects, we performed a second experiment where we tested males and females separately and used focal animals that had been allowed to mate or had been kept as virgins prior to the cohabitation treatment (see electronic supplementary material, figure S2). We concentrated on the 18 h time point, where *Hsp83* expression differences were previously found to be strongest (figure 1), and two treatment groups: N^N^ and N^W^. In addition to *Hsp83*, which codes for the more well-known cytosolic protein of the HSP90 group and is the homologue of *Drosophila Hsp83* [35,53,54], we included the more recently described paralogue *Hsp90*, which codes for an endoplasmatic reticulum-based HSP90 protein [34]. We predicted that *Hsp90* would behave similarly to *Hsp83.* Our data confirm and support the results of the first experiment, showing a persistent and highly significant reduction of *Hsp83* expression in both males and females from the N^W^ group relative to the N^N^ group, which was independent of mating (figure 3). Intriguingly, *Hsp90* showed exactly the same pattern as *Hsp83*. The increased statistical power of this experiment showed that the downregulation of *Imd* due to social transfer of immunity was restricted to females. Furthermore, we confirmed the downregulation of *Hsp68*. Interestingly, it has previously been hypothesized that such HSP70 proteins might also serve a capacitor function [11].

## 4. Conclusion

Taken together, we found persistent downregulation of two Hsp90 group genes in animals where the only treatment was to place them in a group with wounded conspecifics. We could exclude that this effect resulted from changed mating behaviour of the wounded conspecifics, and thus consider it likely that it might represent an adaptive response to the perceived riskiness of the environment. The observed reduction (by 50% or less) is moderate and well within the range that was shown to be biologically relevant in terms of release of cryptic genetic variation both in the laboratory and in natural populations of *Drosophila* [13,16,18]. Our studies provide an important proof of principle that Hsp90 gene expression, and therefore potentially evolvability, could be regulated by an organism in response to environmental cues. It is noteworthy that we recently also observed a trend for reduced expression of *Hsp83* across generations as a consequence of trans-generational immune priming in the same beetle species [55]. Finally, linking Hsp90 expression to social cues derived from wounding represents an ecologically relevant situation [56], since wounding can be associated with infection and thus potentially situations that demand for fast coevolutionary change. This is reminiscent of arguments for the benefit of sexual reproduction for host–parasite coevolution [57]. It remains to be shown how the relevant signals are perceived by the organism, and whether the reduced expression of HSPs facilitates adaptation in this species, be it through the release of cryptic genetic variation or the mobilization of transposons.

## Supplementary Material

Additional Figures and Tables.pdf

## Data accessibility

The raw data supporting the results of this manuscript are deposited in the Dryad digital repository: http://dx.doi.org/10.5061/dryad.60f62.
