# Supplementary material for: Downregulation of the evolutionary capacitor Hsp90 is mediated by social cues
Source: Proc Biol Sci. 2015 Nov 22;282(1819):20152041. doi: 10.1098/rspb.2015.2041 (PMC4685818; doi:10.1098/rspb.2015.2041)
Supplement: Additional Figures and Tables.pdf [file rspb20152041supp1.pdf]

Additional figures & tables

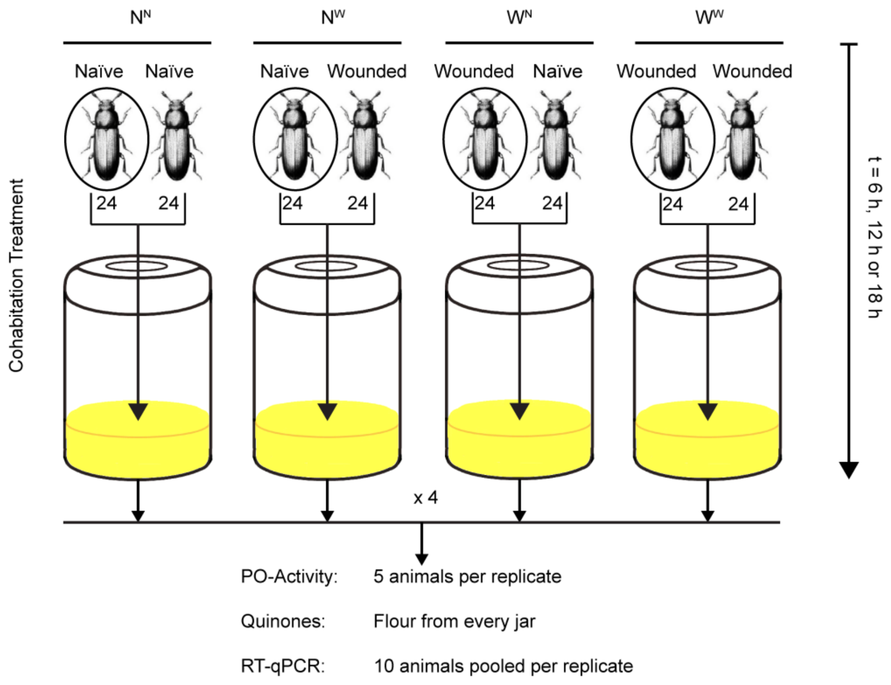

Figure S1 | Setup for experiment 1 to investigate the response of naïve focal *T. castaneum* cohabitated with wounded beetles at different time points. One week old virgin adult beetles were randomly assigned to one of the following treatment groups: 1. naïve focal with naïve non-focal (N^N) 2. naïve focal with wounded non-focal (N^W) 3. wounded focal with naïve non-focal (W^N) and 4. wounded focal with wounded non-focal (W^W). For each treatment 24 focal (circled) and 24 cohabitating (i.e. non-focal) beetles were used and 3 cohabitation times (6, 12 and 18 h) were independently tested. Each treatment / cohabitation time combination had four replicates resulting in a total of 2304 beetles (48 beetles x 4 treatments x 3 cohabitation times x 4 replicates). After cohabitation focal beetles were randomly assigned to evaluate either PO activity or were used for RT-qPCR to determine expression of heat shock protein, stress and immune genes. The flour on which the beetles had been for the respective time periods was used to determine hydroquinone and benzoquinone levels.

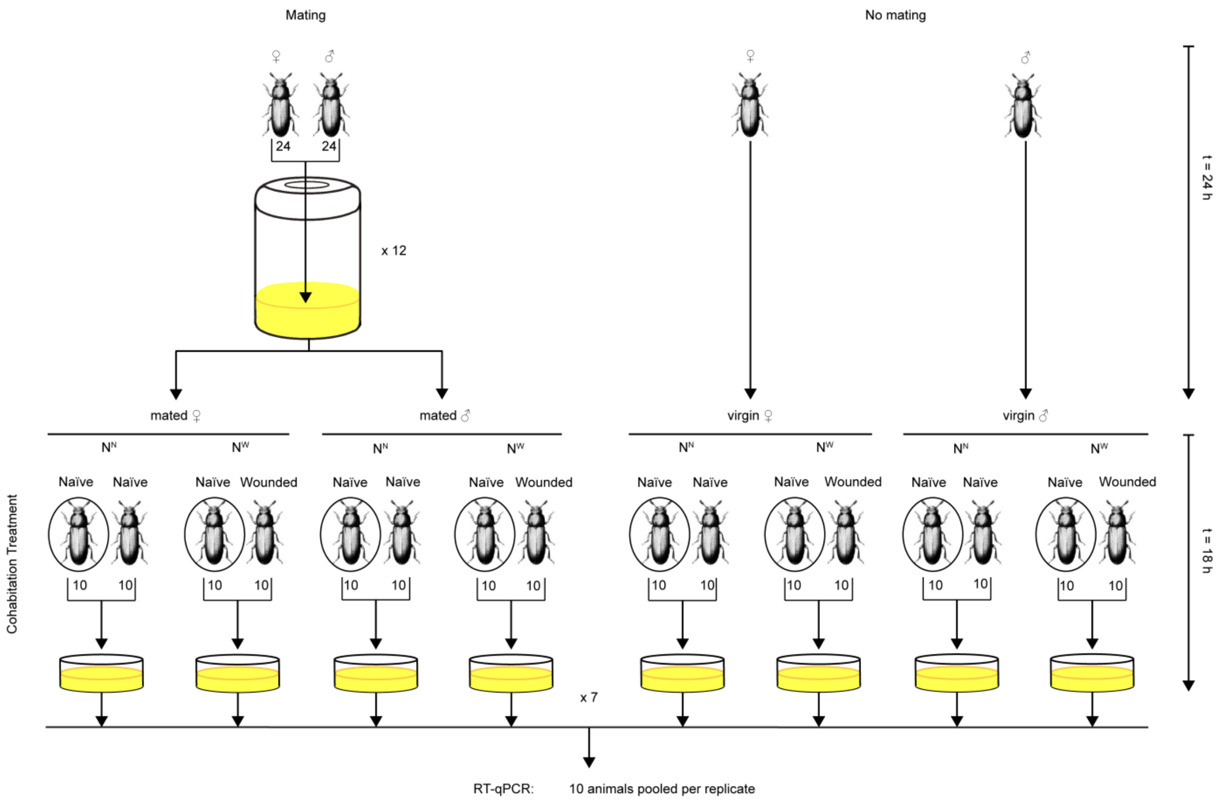

Figure S2 | Setup for experiment 2 to investigate the response of naïve focal *T. castaneum* cohabitated with wounded conspecifics depending on their mating status and sex. One week old virgin adult beetles were randomly assigned to two mating statuses: 1. Mating and 2. No mating. For "Mating" 24 females (♀) and 24 males (♂) were kept together for 24 h under standard conditions. For "No mating" beetles remained individualised for an additional 24 h. Afterwards beetles were separated according to sex and within their mating status and sex group assigned to one of the following treatments: 1. naïve focal beetles with naïve non-focal beetles (N^N), 2. naïve focal beetles with wounded non-focal beetles (N^W). For each treatment 10 focal (circled) and 10 cohabitating (i.e. non-focal) beetles were used and one cohabitation time (18 h) was tested. Each treatment / cohabitation time / mating status and sex combination was replicated 7 times resulting in a total of 1120 beetles (20 beetles x 2 treatments x 1 cohabitation time x 2 mating statuses x 2 sexes x 7 replicates). After cohabitation all focal beetles of each treatment were used to measure the expression of selected stress and immune genes via RT-qPCR.

Table S1. Primer-sequences with respective efficiencies (E) for RT-qPCR. ↓ in primer sequences indicate where RT-qPCR primers cross exon-exon boundary.

Gene Symbol	Name	NCBI Accession Number	E / Fragment length bp	5' – 3' primer sequence	Primer origin
Att2	*Attacin 2*	TC007738	1.94 / 163	F: CAAACGACCAAAG↓GGAAACTA<br>R: CTTCCTCCAAGCAAAGTTGG	This study
Col1	*Coleoptericin 1*	TC005093	1.97 / 120	F: TTTGGCACTTTTTGCACTTG<br>R: GGGATGTCCTGTTCTACGGA	This study
Imd	*Immune deficiency*	TC010851	1.97 / 129	F: CCTCCAAGGGATGAAGTCAA<br>R: ACTGGCAAAAG↓CAGATGGTC	Zou et al, 2007
Hsp83	*Heat shock protein 83*	TC014606	2.00 / 107	F: CGCAGTTCATTGGCTATCCC<br>R: AGGAGGAAGAAGGCGAAGAC	This study
Hsp90	*Heat shock protein 90*	TC012185	1.95 / 128	F: TTGTGGTGTCACGTTTGTGC<br>R: TCTGCTCGTGTGTATCCGATT	This study
Hsp68	*Heat shock protein 68*	TC009706	1.85 / 140	F:CCTATTCCTGCGTCGGAGTC<br>R: GGCAACTTGGTTCTTGGCAG	This study
Thau	*Thaumatin*	TC000517	1.96 / 129	F: ATGGTTGCT↓ATCGAGCCGC<br>R: AACCCCGTTGCCATTTCTGA	This study
CytP450	*Cytochrome P 450*	TC010423	1.91 / 120	F: GTTTGTACCC CTCGGTGCCGTTCTA<br>R: GGTCCCGGTGGATGCCGTAAGCGAAA	This study
Rp49	*Ribosomal protein 49*	TC006106	1.95 / 132	F: TTATGGCAAACTCAAA↓CGCAAC<br>R:GGTAGCATGTGCTTCGTTTTG	Konopova & Jindra, 2007
RpL13a	*Ribosomal protein L13a*	TC013477	2.00 / 186	F: GGCCGCAAG↓TTCTGTCAC<br>R:GGTGAATGGAGCCACTTGTT	This study

References:

Zou Z, Evans JD, Lu Z, Zhao P, Williams M, Sumathipala N, et al. Comparative genomic analysis of the *Tribolium* immune system. Genome Biol. 2007;8:R177-R.

Konopova B, Jindra M. Juvenile hormone resistance gene Methoprene-tolerant controls entry into metamorphosis in the beetle *Tribolium castaneum*. Proc Natl Acad Sci U S A. 2007;104:10488-93.
